# Designing Silver Nanoparticles for Detecting Levodopa (3,4-Dihydroxyphenylalanine, L-Dopa) Using Surface-Enhanced Raman Scattering (SERS)

**DOI:** 10.3390/s20010015

**Published:** 2019-12-18

**Authors:** Rafael Jesus Gonçalves Rubira, Sabrina Alessio Camacho, Cibely Silva Martin, Jorge Ricardo Mejía-Salazar, Faustino Reyes Gómez, Robson Rosa da Silva, Osvaldo Novais de Oliveira Junior, Priscila Alessio, Carlos José Leopoldo Constantino

**Affiliations:** 1 School of Technology and Applied Sciences, São Paulo State University (UNESP), Presidente Prudente 19060-900 SP, Brazil; cibely.martin@unesp.br (C.S.M.); priscila.alessio@unesp.br (P.A.); carlos.constantino@unesp.br (C.J.L.C.); 2 School of Sciences, Humanities and Languages, São Paulo State University (UNESP), Assis 19806-900 SP, Brazil; sabrina.alessio@unesp.br; 3 National Institute of Telecommunications (Inatel), Santa Rita do Sapucaí 37540-000 MG, Brazil; jrmejia@inatel.br; 4 São Carlos Institute of Physics, University of São Paulo (USP), P.O. Box 369, São Carlos 13560-970 SP, Brazil; faustino.reyes@correounivalle.edu.co (F.R.G.); robsilva31@iq.unesp.br (R.R.d.S.);

**Keywords:** L-Dopa, SERS, Ag nanoparticles, multidimensional projections

## Abstract

Detection of the drug Levodopa (3,4-dihydroxyphenylalanine, L-Dopa) is essential for the medical treatment of several neural disorders, including Parkinson’s disease. In this paper, we employed surface-enhanced Raman scattering (SERS) with three shapes of silver nanoparticles (nanostars, AgNS; nanospheres, AgNP; and nanoplates, AgNPL) to detect L-Dopa in the nanoparticle dispersions. The sensitivity of the L-Dopa SERS signal depended on both nanoparticle shape and L-Dopa concentration. The adsorption mechanisms of L-Dopa on the nanoparticles inferred from a detailed analysis of the Raman spectra allowed us to determine the chemical groups involved. For instance, at concentrations below/equivalent to the limit found in human plasma (between 10^−7^–10^−8^ mol/L), L-Dopa adsorbs on AgNP through its ring, while at 10^−5^–10^−6^ mol/L adsorption is driven by the amino group. At even higher concentrations, above 10^−4^ mol/L, L-Dopa polymerization predominates. Therefore, our results show that adsorption depends on both the type of Ag nanoparticles (shape and chemical groups surrounding the Ag surface) and the L-Dopa concentration. The overall strategy based on SERS is a step forward to the design of nanostructures to detect analytes of clinical interest with high specificity and at varied concentration ranges.

## 1. Introduction

Colloidal metallic nanoparticles have been used in various areas, including nanobiotechnology [1,2], cancer diagnosis [3], drug delivery [4,5], and sensing [6,7,8]. Key features of these nanoparticles are the experimental simplicity of their synthesis and possible tuning of plasmonic properties by varying their size, shape and dielectric constant. For surface-enhanced Raman Scattering (SERS), in particular, enhancements up to 6 orders of magnitude can be reached [9,10]. Since adsorption of the analyte on the nanoparticles is required for SERS, incorporating the analyte into the colloidal suspension is advantageous [10], as exemplified in the detection of interleukin-6 with an Au/Ag nanoshell colloid in comparison with quasi-spherical Au nanospheres [11]. Analogously, the adsorption of the herbicide atrazine (ATZ) on Ag nanospheres (AgNP) made it possible to detect picomolar concentrations of ATZ using SERS, whose intensity was higher in solution than in a cast film [7]. This also means that the orientation of ATZ molecules on AgNP is crucial for this ultrasensitive analysis. One may infer that signal enhancement for analytes can be obtained by tailoring the size and shape of the nanoparticles [12,13,14], in addition to the conformation of the analyte adsorbed as in the detection of B-complex vitamins in pharmaceutical samples [15].

Such control in nanoparticle shape is afforded through various synthetic routes [16,17,18], then allowing the Localized Surface Plasmon Resonances (LSPR) to be tuned according to the region of best response (excitation) of the analyte [14,18,19]. For example, the near electromagnetic field is higher in nanoprisms (AgNPR), nanostars (AgNS) and nanorods (AgNR) than on spherical nanoparticles (AgNP) [18,19], and the LSPR may be wider to allow excitation down to the near infrared (IR) [18,19,20]. Indeed, Rycenga et al. [21] reported higher SERS activity for three molecules adsorbed on Ag nanocubes (AgNC) than on AgNP due to the wider LSPR for AgNC. Izquierdo-Lorenzo et al. [18] found that AgNPR were more efficient than AgNP for detecting aminoglutethimide used in sport doping owing to field enhancement in the corners of AgNPR, forming interstitial junctions, which are called “hot spots”.

The adsorption (and orientation, as a consequence) of an analyte can in principle be controlled by: (a) the affinity between analyte and metal; (b) charge of the analyte, which can be modulated via pH; (c) size and shape of the nanoparticles, and (d) metallic surface, which may contain residual ions from the colloidal synthesis [9,10,22]. In order to exploit these dependences for reaching high sensitivity, one may also need to understand the mechanisms in SERS detection, especially as deleterious factors may appear. For instance, SERS analysis may be hampered by changes in the SERS spectra induced by the way analytes are oriented on the nanoparticles [23]. Examples include cases in which the SERS spectrum of the analyte may deviate from its corresponding Raman spectrum [24,25], the SERS spectra may be irreproducible in multiple measurements with identical samples [26], and unknown bands may appear which cannot be assigned to those of the Raman spectra [27,28]. One possible way to identify the mechanisms of analyte adsorption is to determine the chemical groups involved in the interactions between analyte and nanostructures [9,29]. Ag nanoparticles are known to establish interactions with compounds containing nitrogen atoms [30,31,32]. This has been shown in studies where Ag nanospheres (AgNP), nanoplates (AgNPL) and nanostars (AgNS) were employed to detect and quantify dyes [33,34,35], herbicides [36,37], and drugs [18,38,39].

In this paper, we further investigate the SERS mechanisms by comparing detection data with three different shapes of Ag nanoparticles (nanostars, AgNS; nanospheres, AgNP; nanoplates, AgNPL) for the analyte levodopa (3,4-dihydroxyphenylalanine, L-Dopa), a drug of the amino acids family used to treat patients with Parkinson’s disease. This choice was made owing to the medical importance of L-Dopa and the current need for higher sensitivity in its detection in body fluids. L-Dopa is rapidly absorbed to reach the central nervous system, where it is metabolized to dopamine, a neurotransmitter [40,41]. Under normal conditions, the concentration of L-Dopa is typically 10^−8^ mol/L and 10^−7^ mol/L in plasma and urine, respectively [42], but these levels vary considerably in patients with Parkinson’s disease. L-Dopa has been detected with several methods, with varying limits of detection (LOD). A LOD of 2.3 × 10^−5^ mol/L was obtained with spectrophotometry [43], while values of 2.9 × 10^−8^ mol/L [44], 3.0 × 10^−8^ mol/L [45], and 10^−6^ mol/L [46,47,48] were obtained with fluorescence spectroscopy, flow-injection amperometry, and electrochemical analysis, respectively. However, the spontaneous polymerization of L-Dopa represents an obstacle for its detection, requiring more sensitive and simple techniques. SERS seems a suitable alternative, as it has already been employed to detect analytes containing catechol and amino groups, such as catecholamines [49,50], and neurotransmitters [51]. However, both pH and the substituent on the ethylamine chain can influence the SERS signal [49,52]. Herein, in addition to showing the possibility of detecting L-Dopa at low concentration with SERS, we also determine how both nanoparticle shape and L-Dopa concentration affect the adsorption of L-Dopa molecules. Furthermore, cyclic voltammetry was used to compare the adsorption process of L-Dopa with other analytical techniques. In SERS, molecular orientation on metal nanoparticle depends on adsorption mechanism of the analyte, while in electrochemistry the adsorption depends on the surface area.

## 2. Experimental Section

### 2.1. General Information

Silver nitrate (AgNO_3_, MW = 169.88 g/mol), hydroxylamine solution (NH_2_OH, MW = 33.03 g/mol, 50% w/v), hydroxylamine hydrochloride (NH_2_OH·HCl, MW = 69.49 g/mol), sodium borohydride (NaBH_4_, MW = 37.83 g/mol) and sodium citrate (C_6_H_5_Na_3_O_7_ 2H_2_O, MW = 294.10 g/mol) were acquired from Sigma-Aldrich (Cotia, Brazil). Potassium bromide (KBr, MW = 119.00 g/mol) and sodium hydroxide (NaOH, MW = 40.00 g/mol) were purchased from ACP while hydrogen peroxide (H_2_O_2_, MW = 34.01 g/mol, 50% wt/vol) was obtained from Fisher Scientific (Suwanee, GA, USA). All chemicals were used without further purification. Ultrapure water with a resistivity of 18.2 MΩ.cm and pH 5.6, from a Simplicity model Milli-Q system (Merck, Darmstadt, Germany), was used in the synthesis of Ag colloidal nanoparticles. All glassware was cleaned with a sulfochromic solution and rinsed thoroughly with ultrapure water. The target molecule 3,4-dihydroxyphenylalanine (L-Dopa, C_9_H_11_O_4_N, MW = 197.19 g/mol) was purchased from Sigma-Aldrich. Potassium chloride (KCl) from Sigma-Aldrich was used as supporting electrolyte in the electrochemical measurements. All chemicals used have analytical grade with purity higher than 99%. The UV-Vis extinction spectra of AgNS, AgNP and AgNPL colloids and L-Dopa solution were recorded from 200 to 1100 nm using a model Cary 50 spectrophotometer (Agilent Technologies, Santa Clara, CA, USA). Transmission electron microscopy (TEM) images of AgNS, AgNP and AgNPL were recorded with a JEM-1400 transmission electron microscope (JEOL, Peabody, MA, USA) equipped with an Orius SC1000 camera (Gatan Inc., JEOL, Peabody, MA, USA) with a 0.2 nm lattice resolution and a magnification range from ×200 to ×1,200,000.

### 2.2. Synthesis of Ag Nanoparticles

#### 2.2.1. Ag Nanostars (AgNSs)

The AgNS colloid was synthesized according to Garcia-Leis et al. [19] using hydroxylamine and sodium citrate reduction [53]. A mixture with 500 µL of NH_2_OH (6 × 10^−2^ mol/L) and 500 µL of NaOH (5 × 10^−2^ mol/L) was subjected to magnetic stirring followed by addition of 9 mL of AgNO_3_ (1 × 10^−3^ mol/L). The resulting suspension became brown, and stirring was kept for 5 more min. Then, 10 µL of sodium citrate (1% w/v) were poured into the suspension kept under stirring for 15 min, resulting in a colloidal suspension of AgNS with dark gray color.

#### 2.2.2. Ag Nanospheres (AgNPs)

The colloidal suspension of AgNP was synthesized via hydroxylamine hydrochloride reduction as reported by Leopold and Lendl [54]. An aqueous solution was prepared under vigorous magnetic stirring with 4.5 mL of NaOH (0.1 mol/L) and 5 mL of NH_2_OH.HCl (43.3 × 10^−3^ mol/L). Then, 90 mL of AgNO_3_ (1.2 × 10^−3^ mol/L) were quickly added to the aqueous solution, with magnetic stirring being kept for 5 more min to obtain the AgNP colloidal suspension.

#### 2.2.3. Ag Nanoplates (AgNPLs)

Triangular AgNPLs were prepared according to the methodologies proposed by Cathcart et al. [55] and Izquierdo-Lorenzo et al. [18]. 40 mL of an aqueous suspension containing sodium citrate (2.4 × 10^−3^ mol/L), AgNO_3_ (1.2 × 10^−4^ mol/L), H_2_O_2_ (2.6 × 10^−2^ mol/L) and KBr (6.5 × 10^−7^ mol/L) were kept for 30 min in a cold bath (~4 °C) without stirring. The flask was then removed from the cold bath mixed with 480 µL of a freshly prepared NaBH_4_ solution (0.1 mol/L) and brought to vigorous magnetic stirring. The suspension became pale yellow immediately due to the formation of Ag seeds, and the synthesis was finished after 5 min.

### 2.3. SERS Measurements

A stock solution of L-Dopa was prepared in ultrapure water at 10^−2^ mol/L, under sonication. The stock solution was diluted in three different colloidal suspensions (AgNS, AgNP and AgNPL) at final concentrations of 10^−3^, 10^−4^, 10^−5^, 10^−6^, 10^−7^ and 10^−8^ mol/L. To obtain the analysis concentration of L-Dopa, the final volume was adjusted to 1 mL using the colloidal suspensions. The SERS spectra were obtained by dripping small droplets of L-Dopa solutions (diluted in Ag colloids) on a holder under the microscope, and the laser focus was adjusted at the air/water interface. For each colloidal suspension (AgNS, AgNP and AgNPL) in presence of L-Dopa with concentration higher than 10^−5^ mol/L, at least 10 spectra were recorded. However, for lower concentrations, from 50 to 100 acquisitions are necessary. The SERS spectra were acquired using a model in-Via micro-Raman system (Renishaw, Wotton Under Edge in Gloucestershire, United Kingdom). The micro-Raman is equipped with a microscope (Leica, Wotton Under Edge in Gloucestershire, United Kingdom) whose 50× microscope objective long lens allows for collecting spectra with ca 1 μm^2^ spatial resolution, a CCD detector and a computer-controlled three-axis-encoded (XYZ) motorized stage to record Raman spectra with a minimum step of 0.1 μm. The spectra were obtained with the laser line at 633 nm, 1800 grooves/mm grating, dielectric filters, and spectral acquisition time of 10 s.

### 2.4. Data Analysis—Multidimensional Projections

The SERS spectra were treated using a projection technique, in which data from a multidimensional space (with various wavenumbers in the SERS spectra) is projected onto a plane according to the similarity (and differences) in the data set. The flexibility of this optimization approach arises from the availability of several cost (or error) functions for placing the graphical markers on the 2D plot. In this study, each SERS spectrum was reduced to a data point positioned with application of the so-called Interactive Document Mapping (IDMAP) technique [56] whose function is defined as:

(1) $$S_{IDMAP}=\frac{\delta (x_{i},x_{j})-\delta_{\min}}{\delta_{\max}-\delta_{\min}}-d(y_{i},y_{j})$$

where *δ* and *d* are the distance functions defined for the original (of SERS spectra) and projected spaces (of graphical markers), and *δ*_min_ and *δ*_max_ are the minimum and maximum distances between the samples. By samples we mean the SERS spectra for each Ag nanoparticle and each L-Dopa concentration.

### 2.5. Electrochemical Measurements

The electrochemical measurements were performed using screen-printed carbon electrodes (SPCE) modified with colloidal suspensions of AgNS, AgNP and AgNPL. The colloidal suspensions were diluted in ultrapure water at 1:4 (v/v) ratio and the SPCE was modified by dropping 30 µL of the diluted colloidal suspensions. The modified SPCE was kept at room temperature for 24 h before the electrochemical measurements, which were performed with a 3-electrode cell: the reference electrode made with Ag/AgCl/3 mol/L KCl, a platinum wire used as counter electrode and the working electrode being the modified SPCE. A µ-autolab potentiostat/galvanostat type III (Metrohm, Herisau, Switzerland) was used to obtain cyclic voltammograms from −0.2 to +0.8 V and a scan rate of 25 mV/s in 10^−6^ mol/L of L-Dopa standard solution containing 0.1 mol/L KCl as supporting electrolyte.

## 3. Results and Discussion

### 3.1. Characterization of the Ag Nanoparticles: AgNS, AgNP and AgNPL

**Figure 1:**
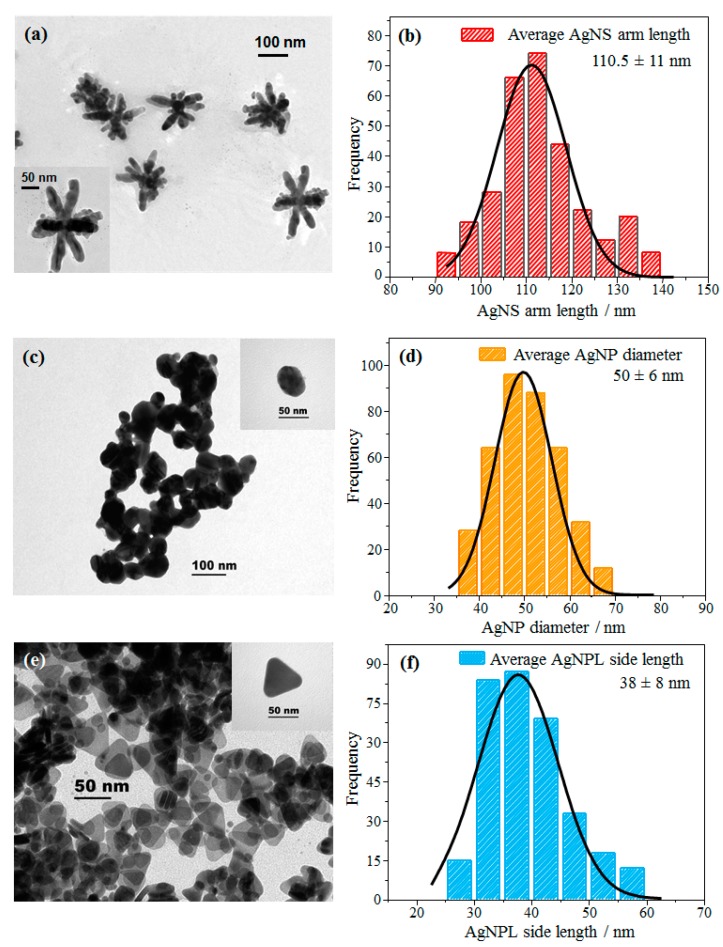
TEM images and histograms of different shapes of Ag nanoparticles: (**a**,**b**) AgNS, (**c**,**d**) AgNP and (**e**,**f**) AgNPL. The insets show microscopy images with higher resolution.

Figure 1 exhibits TEM images and histograms of different shapes of Ag nanoparticles: nanostars (AgNS), nanospheres (AgNP) and nanoplates (AgNPL). For AgNS in Figure 1a,b, the length of the arms (L) is 110.5 nm on average, resulting in diameters of ca. 221 nm. The TEM image shows nanostars with different numbers of arms and a large dispersion for the arm lengths, with a standard deviation of ±11 nm (see the histogram in Figure 1b). According to Garcia-Leis et al. [19] these different morphologies are the result of the low amount of Ag atoms available in the mixture, i.e., the lower the amount of Ag^+^, the shorter the length of the arms in the AgNS colloid, also resulting in nanostars with different numbers of arms. The TEM image of AgNP in Figure 1c shows a predominant spherical shape with an average diameter (D) of 50 nm (see the histogram in Figure 1d), consistent with Leopold and Lendl [54] and Canamares et al. [57]. The nanospheres have a standard deviation in average diameter of ± 6.0 nm (see Figure 1d), indicating a more monodisperse size distribution than for AgNS and AgNPL. For AgNPL, the TEM image in Figure 1e points to a colloidal suspension mainly formed by triangular nanoplates, with an average size of side lengths (L) of 38 nm (histogram in Figure 1f). The large side length distribution is related to different sizes of AgNPL in the colloid. Also, triangular morphologies with non-sharp corners are seen in the TEM image (Figure 1e). Izquierdo-Lorenzo et al. [18] reported similar morphologies with the same methodology to synthesize AgNPL. AgNP are also present in the AgNPL colloidal suspension, although at lower concentration than AgNPL. The high amount of citrate (citrate/Ag^+^ ratio (>1)) used in the synthesis keeps the concentration of nanospheres low, resulting in a majority of nanoplates [58].

**Figure 2:**
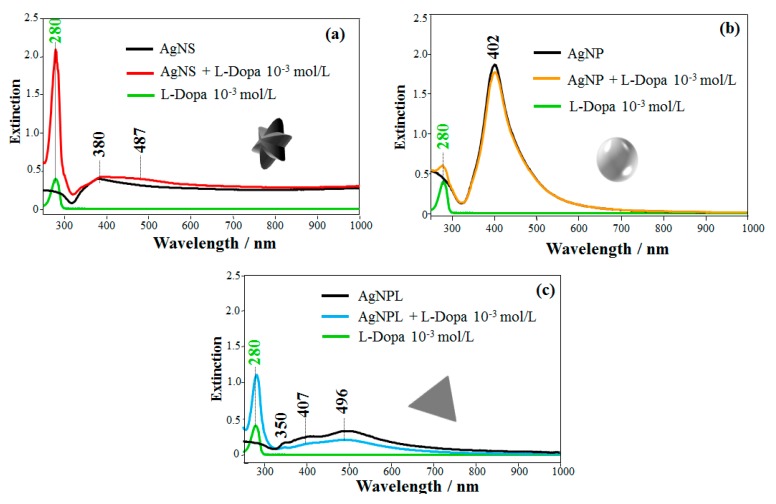
Extinction spectra of L-Dopa solution (10^−3^ mol/L) in colloidal suspensions of Ag nanoparticles with three shapes: (**a**) AgNS + L-Dopa, (**b**) AgNP + L-Dopa and (**c**) AgNPL + L-Dopa.

The UV-Vis spectra of L-Dopa solution (10^−3^ mol/L) and of Ag nanoparticles with different shapes (AgNS, AgNP, and AgNPL) are displayed in Figure 2. L-Dopa exhibits an absorption band at 280 nm assigned to π-π* transitions from the benzene ring, characteristic of catecholamines [59]. Figure 2a displays the extinction spectrum of AgNS with a maximum plasmon resonance at 380 nm and a tail at longer wavelengths, which can be related to the absorption and scattering of the different morphologies of AgNS [19,60,61], consistent with the TEM image in Figure 1a. For AgNP, the maximum plasmon resonance at 402 nm in Figure 2b agrees with the values reported for AgNP synthesized via hydroxylamine reduction [18,57]. The extinction spectrum of AgNPL in Figure 2c displays three bands at 350, 407 and 496 nm, assigned to the out-of-plane quadrupole, in-plane quadrupole, and in-plane dipole plasmon resonance modes of triangular nanoplates, respectively [18,62,63,64].

The extinction spectra of Ag nanoparticles containing L-Dopa (10^−3^ mol/L) are also presented in Figure 2. For the mixture of AgNS + L-Dopa (Figure 2a), the strongest absorption band appears at 280 nm, assigned to π-π* transitions of L-Dopa. Comparing this spectrum with the AgNS spectrum, the band at 380 nm assigned to the quadrupole of AgNS [18,63,65,66] is broader (FWHM from 68 nm in AgNS to 79 nm in AgNS + L-Dopa) and a band at 487 nm appeared, suggesting aggregation of the nanoparticles [10,66,67]. On the other hand, for AgNP + L-Dopa mixture in Figure 2b, no changes were observed in the plasmon extinction of the AgNP, which indicate no aggregation. The plasmon extinction bands for AgNPL + L-Dopa at 350, 407 and 496 nm in Figure 2c had their intensities decreased, and the FWHM of the 496 nm band, assigned to in-plane dipole plasmon mode, changed from 176 nm in AgNPL to 220 nm in AgNPL + L-Dopa, which may also suggest aggregation [66]. Garcia-Ramos et al. [18] also noted a decrease in the plasmon extinction for AgNPL in the presence of aminoglutethimide (AGI) owing to adsorption of AGI on AgNPL and aggregation of the colloidal system. These changes in the plasmon bands suggest a change in the dielectric constant of the medium (colloid + analyte). Therefore, AgNS and AgNPL were more affected by L-Dopa, which induced colloidal aggregation denoted by changes in the extinction spectra. According to Rodríguez-Lorenzo et al. [66], aggregation of AgNS results in a higher number of hot spots than for AgNPL aggregation, thus leading to higher enhancement factors. The changes in the extinction spectra could also be induced by interaction between L-Dopa and the reducing agents used in the colloidal synthesis, which differ for the three colloidal suspensions. Another possibility for such changes could be the alteration in the polarization state of scattered light owing to breaking the dipolar symmetry for trimers, quadrimers and higher-order symmetry arrangements [68,69].

### 3.2. SERS Measurements for Detecting L-Dopa

**Figure 3:**
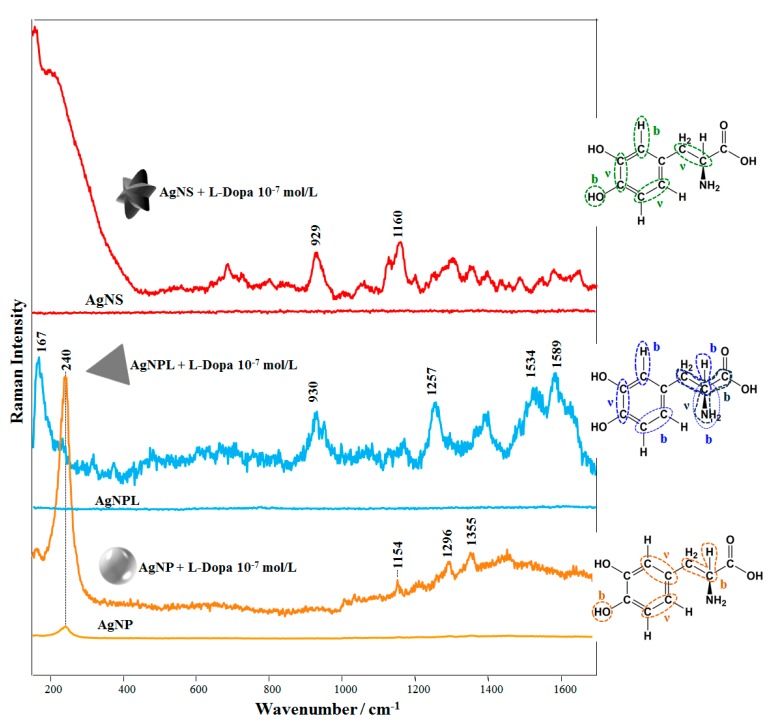
SERS spectra of L-Dopa solutions in colloidal suspensions of AgNPL, AgNP, and AgNS (final concentration of L-Dopa: 10^−7^ mol/L). The insets depict molecular structures of L-Dopa with the vibrational modes related to the most enhanced SERS bands. The symbols “b” and “ν” refer to bending and stretching modes, respectively. At 10^−7^ mol/L of L-Dopa, the UV-Vis extinction spectra in Figure 2 have kept their profiles. Laser line at 633 nm. All the spectra were obtained under the same experimental conditions and are plotted without smoothing or offset correction. The Raman spectra of the colloidal suspensions (without L-Dopa) are given at the same intensity scale of the corresponding L-Dopa SERS spectra (10^−7^ mol/L) to facilitate comparison.

Figure 3 shows the SERS spectra recorded for L-Dopa solutions (final concentration: 10^−7^ mol/L) for the three colloidal suspensions (AgNS, AgNP, and AgNPL). The SERS spectra for L-Dopa at low concentrations (10^−7^ to 10^−5^ mol/L) were not straightforward to obtain. At pH 6.0 L-Dopa is in the zwitterionic form, which can hamper its adsorption on the Ag nanoparticle surface, leading to a low-intensity SERS signal. This has been reported for the zwitterionic form of L-Dopa using solid Ag substrates [49]. In our case, the possibility of obtaining an acceptable SERS profile from L-Dopa at 10^−7^ mol/L as shown in Figure 3 is only ca. 5%. For instance, under this condition the signal-to-noise ratio (SNR) for the SERS spectra (n = 5) was 2.4 (at 930 cm^−1^), 2.7 (at 929 cm^−1^), and 3.3 (at 1154 cm^−1^), using AgNPL, AgNS, and AgNP, respectively. More details about SNR calculation and spectrum acquisition are described in Table S1 and Figure S1 (Supporting Information). These SNR values are consistent with the poor adsorption of L-Dopa on the Ag surface, responsible for fluctuations of the L-Dopa SERS signal, as also observed with diluted solutions owing to changes in the analyte surroundings [70]. In fact, these fluctuations are similar to the “blinking effect” reported for SERS at the single molecule regime when molecules are physically adsorbed on the nanoparticle [71]. Nevertheless, the enhanced bands can be identified by comparing them with the Raman spectra of the colloidal suspensions (without L-Dopa) and with the Raman spectra of the L-Dopa powder. The Raman spectra of AgNP, AgNS and AgNPL colloidal suspensions are also shown as reference in Figure 3. Extended Raman spectra from colloidal suspensions (without L-Dopa) are shown in Figure S2 (Supporting Information), revealing only the bands at 240 cm^−1^ (Ag-Cl stretching) and 3200 cm^−1^ (water band) [35,36]. The aqueous solution of L-Dopa at 10^−1^ mol/L showed no Raman bands, while the Raman spectrum from L-Dopa powder and its assignment (vibrational bands) are given in Figure S3 and Table S2 (Supporting Information). The two bands with highest enhancements in the SERS spectrum of AgNS + L-Dopa are 929 and 1160 cm^−1^ assigned to stretching of C-C from Dopa ring + stretching of C-C and in-plane bending of C-H + stretching of C-C from Dopa ring + out-of-plane bending of O-H [72], respectively. For the SERS spectrum of AgNP + L-Dopa, the most intense band at ca. 240 cm^−1^ is assigned to Cl ions on the AgNP surface (AgNP were synthesized by reduction with hydroxylamine hydrochloride) [35] (Figure S1). The two other bands with large enhancement at 1296 and 1355 cm^−1^ are attributed to stretching of C-C from the L-Dopa ring + in-plane bending of C-C-H and stretching of C-C from the L-Dopa ring + out-of-plane bending of O-H [72], respectively. For AgNPL + L-Dopa, the main enhanced bands are at 167, 1257, 1534 and 1589 cm^−1^. They are assigned to in-plane bending of C-C-C + stretching of C-C-N, in-plane bending of C-N-H, C-C-H + stretching of C-C from the L-Dopa ring [68] and C-C stretching + C-H in-plane bending of the L-Dopa ring [72], respectively. The assignment of the main enhanced bands of SERS spectra is given in Table 1 and highlighted in the molecular structures of L-Dopa (insets, Figure 3).

The adsorption mechanism of the analyte can be determined by considering the surface selection rules [9,10,29], where the signal is preferentially enhanced for modes vibrating perpendicularly and as close as possible to the metallic surface. Adsorption of L-Dopa molecules (at 10^−7^ mol/L) for the three colloidal suspensions (AgNS, AgNP, and AgNPL) can be inferred as follows. The most enhanced bands in the SERS spectra AgNS + L-Dopa and AgNP + L-Dopa refer to vibrational modes of the L-Dopa ring and out-of-plane bending of hydroxyl group. The enhancement of these bands suggests that L-Dopa molecules (at 10^−7^ mol/L) are adsorbed on AgNS and AgNP surfaces through the hydroxyl group + L-Dopa ring. A similar adsorption mechanism is noticed for AgNPL + L-Dopa at 10^−7^ mol/L. However, the vibrational modes related to the amino group at 167 and 1257 cm^−1^ indicate an influence of amino group on adsorption. Hence, L-Dopa molecules may be adsorbed on AgNPL through the amino group + L-Dopa ring.

**Figure 4:**
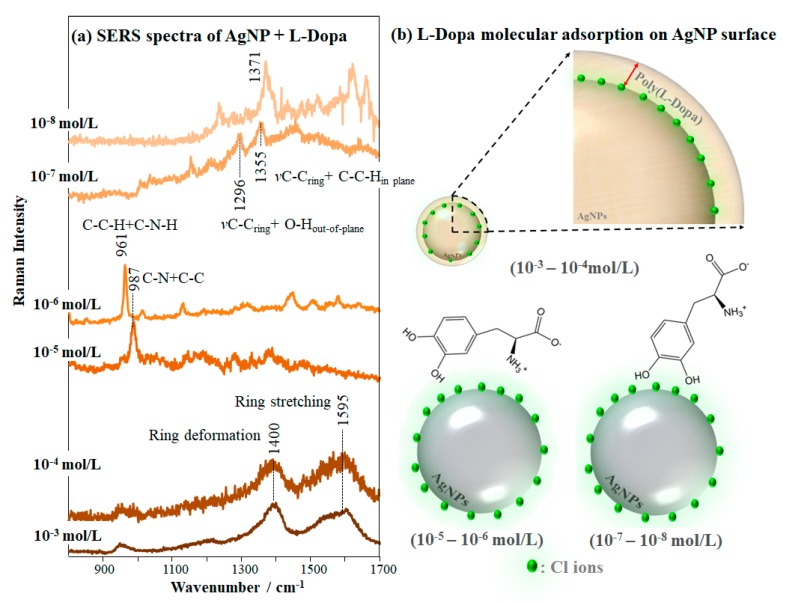
(**a**) SERS spectra of L-Dopa solutions in colloidal suspension of AgNP at concentrations varying from 10^−3^ to 10^−8^ mol/L (laser line at 633 nm), giving the most enhanced bands. The extended SERS spectra are shown in Figure S4. (**b**) Schematic representation of L-Dopa molecular adsorption on the AgNP surface for the different concentrations: at 10^−5^ and 10^−6^ mol/L the L-Dopa competes each other in addition to the Cl ions and adsorbs on AgNP surface through the amino group + L-Dopa ring; at 10^−7^ and 10^−8^ mol/L the L-Dopa competes only with the Cl ions, adsorbing through the hydroxyl group + L-Dopa ring.

The colloidal dispersions prepared with L-Dopa and all Ag nanoparticles studied here exhibited SERS bands from 800 to 1700 cm^−1^, from which we noted that L-Dopa adsorption mechanism depends on the type of Ag nanoparticles, as shown in Figure 3, and on the L-Dopa concentration. The concentration affects the adsorption mechanism of L-Dopa on the Ag surface and the fluctuation of the SERS signal decreases with increasing L-Dopa concentration. We determined the concentration dependence of L-Dopa adsorption mechanism for the three AgNP colloidal systems studied here. The SERS spectra at different L-Dopa concentrations in AgNP colloid are displayed in Figure S4 (Supporting Information) and a zoomed view, from 800 to 1700 cm^−1^, is shown in Figure 4a. The two broad bands at 1400 and 1595 cm^−1^ for AgNP + L-Dopa 10^−3^ and 10^−4^ mol/L are assigned to deformation and stretching modes of the rings, respectively [75]. These broad bands indicate polymerization of L-Dopa molecules, forming polymer chains surrounding the AgNP [73,74]. High concentrations of L-Dopa favor chemical oxidation to quinone, rather than to dopachrome, which polymerizes spontaneously. Because of this property, L-Dopa can also reduce the remaining Ag^+^ of the colloid, increasing the polymerization rate [76,77]. At 10^−5^ mol/L and 10^−6^ mol/L the L-Dopa molecules exhibit the most enhanced bands at 987 cm^−1^, assigned to stretching of C-N + C-C, and at 961 cm^−1^ due to in-plane bending of C-C-H + C-N-H [72]. Therefore, L-Dopa molecules may be adsorbed through amino group + L-Dopa ring on the AgNP surface. On the other hand, at 10^−8^ mol/L the L-Dopa molecules present the same adsorption mechanism determined for 10^−7^ and discussed previously (hydroxyl group + L-Dopa ring). The adsorption sites of L-Dopa molecules (L-Dopa rings, especially in the direction of the oxygen atoms, and in close proximity to the nitrogen atoms) have a density of negative charges [78].

In summary, when approaching AgNP, L-Dopa molecules at 10^−7^ and 10^−8^ mol/L compete with Cl^−^ ions for adsorption on the AgNP, adsorbing through the hydroxyl group + L-Dopa ring. At higher concentrations (10^−5^ and 10^−6^ mol/L) the L-Dopa molecules compete with each other in addition to the Cl^−^ ions. Hence, L-Dopa molecules adsorb on AgNP especially through the amino group + L-Dopa ring, with the dipole moment of the C-N bond perpendicular to the AgNP surface. The molecular adsorption mechanisms discussed above and proposed for different concentrations of L-Dopa in AgNP colloidal suspension (from 10^−3^ to 10^−8^ mol/L) are represented in Figure 4b. Furini et al. [36] observed a similar behavior while detecting high concentrations (10^−5^ mol/L) of the herbicide carbendazim with SERS, which were oriented perpendicular to the AgNP surface, adsorbing through the nitrogen atom of the benzimidazole group.

### 3.3. Multidimensional Projections

**Figure 5:**
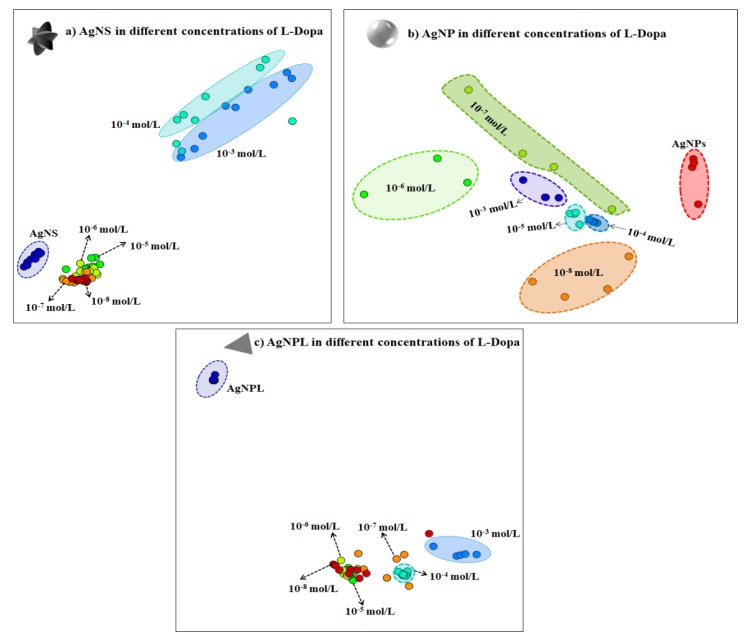
IDMAP multidimensional projection for different concentrations of L-Dopa (from 10^−3^ to 10^−8^ mol/L) in the three colloidal suspensions: (**a**) AgNS, (**b**) AgNP and (**c**) AgNPL. Each circle in the plot represents a whole SERS spectrum. The proximity of the circles indicates the similarity between the data (i.e., similar SERS spectra will lead to circles close to each other).

The performance in detecting L-Dopa with AgNS, AgNP and AgNPL can be inferred by analyzing the SERS sensing data using a multidimensional projection technique. In this procedure, the similarity between SERS spectra is represented by the proximity between the circles in the plot, where each circle represents a whole SERS spectrum (details in Paulovich et al. [79] and Oliveira et al. [80]). IDMAP multidimensional projection has been used in previous works, e.g., to distinguish different concentrations of pesticides in AgNP [7,81] and to detect antigens in a SERS immunoassay platform [82]. When the data from the SERS spectra are projected with the IDMAP technique as in Figure 5, a clear separation of all L-Dopa concentrations down to 10^−8^ mol/L is observed only using AgNP (Figure 5b). For AgNS (Figure 5a) and AgNPL (Figure 5c), an overlapping of the circles is noticed for L-Dopa concentrations down to 10^−5^ mol/L. This behavior can be a consequence of fluctuations on the SERS spectra affecting Raman shift, band shape, bandwidth, and/or relative and absolute intensities for diluted solutions, as discussed previously. A zoomed view of the IDMAP multidimensional projections for the low concentrations is given in Figure S5 (Supporting Information).

### 3.4. Effect of Nanoparticles Shape on Electrochemical Oxidation of L-Dopa

**Figure 6:**
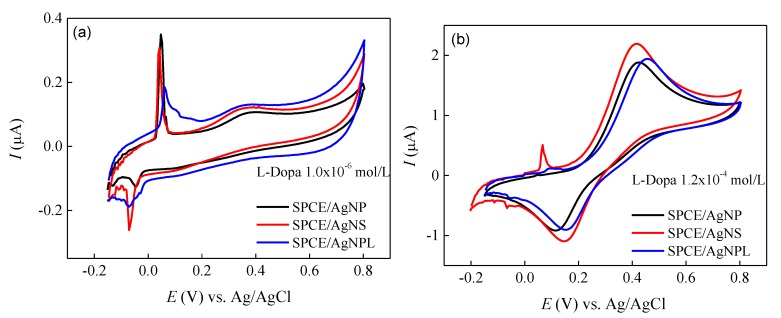
Cyclic voltammetry of SPCE modified with AgNP, AgNS and AgNPL in (**a**) 10^−6^ mol/L and (**b**) 120 × 10^−6^ mol/L of L-Dopa solution. *v* = 25 mV/s.

The results from SERS pointed to AgNP colloidal suspension as the most suitable to detect L-Dopa, probably owing to an increased adsorption of the analyte on the AgNP, in comparison to AgNS and AgNPL. This can be tested with an independent electrochemical method whose response depends on the coating of the nanoparticles by L-Dopa. Here, we first obtained the cyclic voltammograms of screen-printed carbon electrodes (SPCEs) in dispersions of the nanoparticles in a supporting electrolyte. Figure S6 (Supporting Information) shows that in the absence of L-Dopa the onset potential for Ag oxidation in the Ag(0)/Ag(I) redox couple [83] is independent of the nanoparticle shape. In contrast, Figure 6 shows that the Ag oxidation peak decreases with increasing L-Dopa concentration. For 10^−6^ mol/L of L-Dopa (containing 0.1 mol/L KCl solution at pH 5.6), the L-Dopa oxidation to quinone shows no significant current enhancement, and there is no difference in oxidation potential for the different nanoparticle shapes. However, at higher L-Dopa concentrations (120 × 10^−6^ mol/L, Figure 6b), the current of oxidation and reduction of L-Dopa to quinone becomes more intense, and the Ag oxidation peak is almost covered. The well-defined Ag oxidation peak was observed only for SPCE modified with AgNS, which can be related to the nanoparticle shape. This occurs at analyte concentrations high enough to reach complete coverage of the nanoparticles. In our study, the AgNS presents a morphology containing tip protrusions, formed by different numbers of arms and different arm lengths, increasing the contact area, and then the surface is not fully covered by L-Dopa molecules, allowing the oxidation of Ag at ~6 mV. From an electrochemical point of view, the increase in contact area means that AgNS could be applied in electrochemical sensors since defects in Ag nanoparticles can contribute to increase the electrochemical signal [83]. Therefore, in electrochemical sensors a higher sensitivity should be achieved with AgNS, as observed here (see Figure 6). The electrochemical method was not capable of distinguishing L-Dopa concentrations below 10^−6^ mol/L, but this was possible with SERS, as demonstrated above. With SERS, the most sensitive results were obtained with AgNP (and not with AgNS) because sensitivity also depends on the adsorption mechanism of the analyte molecules as they adsorb onto the nanostructures.

## 4. Conclusions

Three shapes of Ag nanoparticles (nanostars: AgNSs, nanospheres: AgNPs, nanoplates: AgNPLs) were designed for detecting L-Dopa within a wide range (from 10^−3^ to 10^−8^ mol/L) using surface-enhanced Raman scattering (SERS). A higher sensitivity for AgNP is suggested using multidimensional projections to analyze the SERS spectra. This was attributed to the distinct adsorption mechanisms of L-Dopa on the nanostructures. In particular, the SERS spectral modifications with the analyte concentration and shape of nanoparticles allowed the analysis of the chemical groups involved in the L-Dopa interactions. In order to confirm the inferences about the adsorption mechanisms, we employed an electrochemical method to detect L-Dopa. The sensitivity in electrochemical detection is higher with AgNS owing to the larger amount of adsorbed L-Dopa, but detection was only possible at higher L-Dopa concentrations. As expected, the electrochemical method was not as sensitive as SERS. One should nevertheless emphasize that only ca. 5% of the SERS measurements at low concentration (10^−7^ mol/L in this case) led to a spectrum with acceptable signal-to-noise ratio. This SERS signal fluctuation is associated with the zwitterionic form of L-Dopa at pH 6.0, for which adsorption on the Ag surface is poor. Significantly, both methods can be applied for detecting L-Dopa in concentrations above 10^−6^ mol/L. The relevance of nanoparticle shape for the two detection methods arises from the additional dependence on the analyte adsorption mechanism (molecular orientation, as consequence) on the nanoparticle in SERS, which does not happen in the electrochemical methods. The procedures related to a detailed analysis of SERS data with determination of chemical groups responsible for adsorption can be extended to other analytes, especially the catecholamines that possess similar structures to L-Dopa.

## Figures and Tables

**Table 1:** Assignments of the SERS bands of L-Dopa solutions (10^−7^ mol/L) in AgNS, AgNP and AgNPL.

AgNS + L-Dopa (cm^−1^)	AgNP + L-Dopa (cm^−1^)	AgNPL + L-Dopa (cm^−1^)	Assignments	Ref.
-	-	167	C-C-C in-plane bending; C-C-N stretching	[73]
929	-	930	C-C stretching of L-Dopa ring; C-C stretching	[73]
1160	-	-	C-H in-plane bending; C-C stretching of Dopa ring; O-H out-of-plane bending	[73,74]
1160	1154	1169	C-H in-plane bending; C-C stretching of L-Dopa ring; O-H out-of-plane bending	[73,74]
-	-	1257	C-N-H and C-C-H in-plane bending; C-C stretching of L-Dopa ring	[73,75]
-	1296	-	C-C stretching of L-Dopa ring; C-C-H in-plane bending	[73]
-	1355	-	C-C stretching of L-Dopa ring; O-H out-of-plane bending	[73,75,76]
-	-	1534	C-H in-plane bending and C-C stretching of L-Dopa ring	[73]
-	-	1589	C-C stretching and C-H in-plane bending of L-Dopa ring	[73,75]

## Supplementary Materials

The following are available online at https://www.mdpi.com/1424-8220/20/1/15/s1, Figure S1: (a) Raman spectra of AgNP colloidal suspensions. (b) SERS spectra of AgNP colloidal suspensions in the presence of 10^−7^ mol/L of L-Dopa showing no active L-Dopa SERS signal. (c) SERS spectra of AgNP colloidal suspensions in the presence of 10^−7^ mol/L of L-Dopa showing the L-Dopa SERS signal, which corresponds to ca. 5% of all spectra recorded. The highlighted band at 1154 cm^−1^ (Figure S1c) was used to calculate SNR (SNR = 3.3). Laser line at 633 nm, Table S1. Data from SERS spectra of L-Dopa at 10^−7^ mol/L used for SNR calculation, Figure S2. Raman spectra of AgNPs, AgNS and AgNPR colloidal suspensions (as reference), Figure S3. Raman spectrum of L-Dopa powder. Laser line at 633 nm, Table S2. Assignments of Raman vibrational bands characteristic of L-Dopa powder, Figure S4. SERS spectra of L-Dopa solutions in colloidal suspension of AgNP at different concentrations (from 10^−3^ to 10^−8^ mol/L). Laser line at 633 nm, Figure S5. Zoom of the IDMAP multidimensional projection for concentrations of L-Dopa (a) down to 10^−5^ mol/L in colloidal suspension of AgNS and (b) down to 10^−4^ mol/L in colloidal suspension of AgNPL. Each circle in the plot represents a whole SERS spectrum. The proximity of the circles indicates the similarity between the SERS spectra, Figure S6a Cyclic voltammetry of SPCE unmodified and modified with AgNP, AgNS and AgNPL in 0.1 mol/L KCl solution. (b) Cyclic voltammetry of SPCE unmodified and modified with AgNS from Figure S6a for better view. *v* = 25 mV/s.
