# Supplementary material for: Designing Silver Nanoparticles for Detecting Levodopa (3,4-Dihydroxyphenylalanine, L-Dopa) Using Surface-Enhanced Raman Scattering (SERS)
Source: Sensors (Basel). 2019 Dec 18;20(1):15. doi: 10.3390/s20010015 (PMC6982777; doi:10.3390/s20010015)
Supplement: Supplementary file 1 [file sensors-20-00015-s001.pdf]

sensors

Supplementary Materials

Article

Designing Silver Nanoparticles for Detecting Levodopa (3,4-Dihydroxyphenylalanine, L-Dopa) Using Surface-Enhanced Raman Scattering (SERS).

Rafael Jesus Gonçalves Rubira [1,*], Sabrina Alessio Camacho [2], Cibely Silva Martin [1], Jorge Ricardo Mejía-Salazar [3], Faustino Reyes Gómez [4], Robson Rosa da Silva [4], Osvaldo Novais de Oliveira Junior [4], Priscila Alessio [1] and Carlos José Leopoldo Constantino [1]

1. School of Technology and Applied Sciences, São Paulo State University (UNESP), Presidente Prudente 19060−900 SP, Brazil; cibely.martin@unesp.br (C.S.M.); priscila.alessio@unesp.br (P.A.); carlos.constantino@unesp.br (C.J.L.C.)
2. School of Sciences, Humanities and Languages, São Paulo State University (UNESP), Assis 19806−900 SP, Brazil; sabrina.alessio@unesp.br (S.A)
3. National Institute of Telecommunications (Inatel), Santa Rita do Sapucaí 37540−000 MG, Brazil; jrmejia@inatel.br (J.R.M.-S)
4. São Carlos Institute of Physics, University of São Paulo (USP), P.O. Box 369, São Carlos 13560−970 SP, Brazil; faustino.reyes@correounivalle.edu.co (F.R.G.); robsilva31@iq.unesp.br (R.R.d.S.); chu@ifsc.usp.br (O.N.O.J)

* Correspondence: rafael.rubira@unesp.br (R.J.G.R)

Received: 15 October 2019; Accepted: 10 December 2019; Published: date

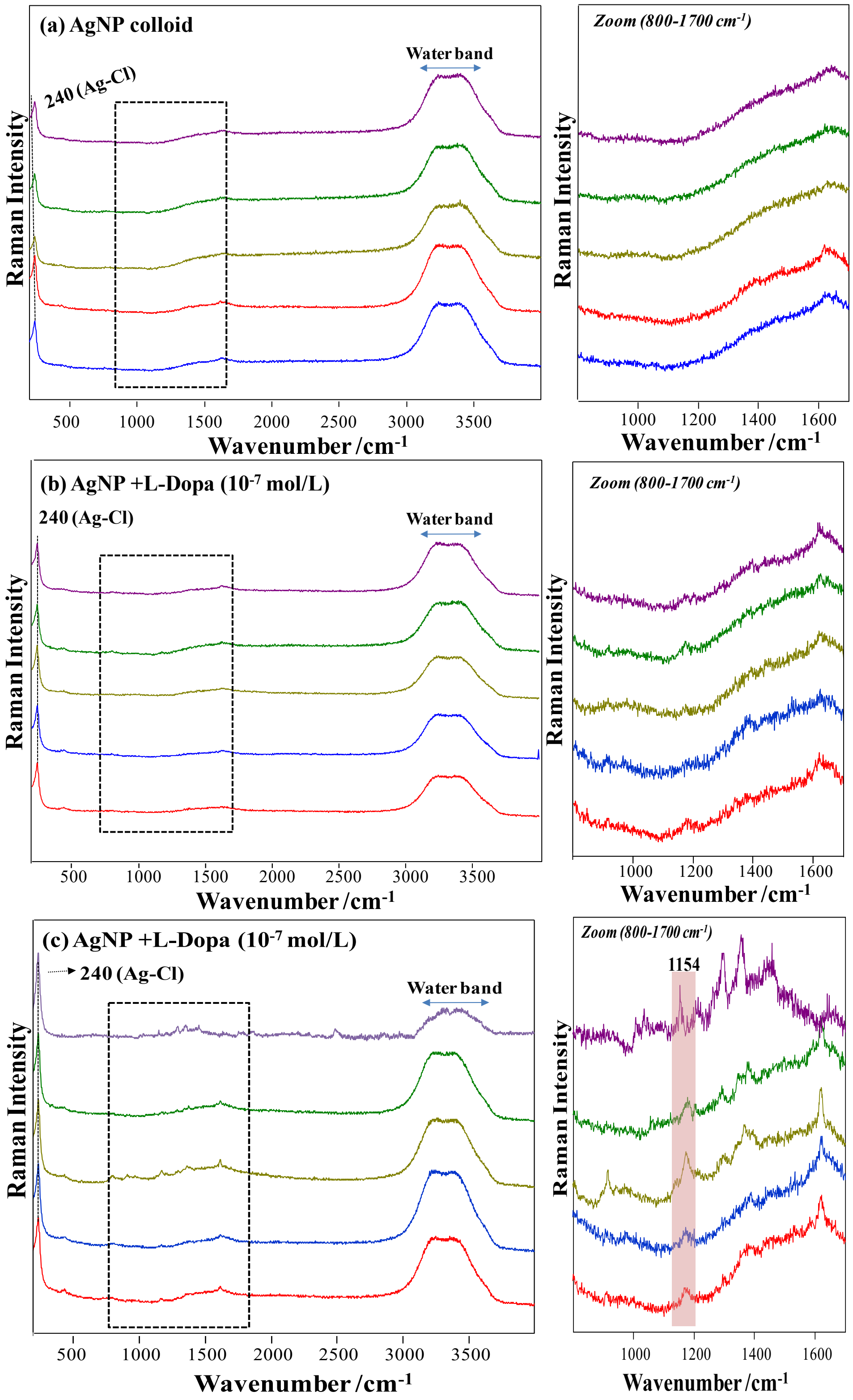

Figure S1. (**a**) Raman spectra of AgNP colloidal suspensions. (**b**) SERS spectra of AgNP colloidal suspensions in the presence of 10^{-7} mol/L of L-Dopa showing no active L-Dopa SERS signal. (**c**) SERS spectra of AgNP colloidal suspensions in the presence of 10^{-7} mol/L of L-Dopa showing the L-Dopa SERS signal, which corresponds to ca. 5% of all spectra recorded. The highlighted band at 1154 cm^{-1} (Figure S1c) was used to calculate SNR (SNR = 3.3). Laser line at 633 nm.

Table S1. Data from SERS spectra of L-Dopa at 10^{-7} mol/L used for SNR calculation.

**Nanoparticles**	AgNPL	AgNS	AgNP
**SERS band (cm^{-1})**	930	929	1154
**Band area**	40583.91	5251.49	10488.93
	23184.21	2757.51	9906.99
	23400.64	2669.15	9891.58
	18939.07	2563.41	9114.99
	14285.25	2489.87	4102.47
**Average (n=5)**	24078.61	3146.29	8700.99
**Standard deviation**	9949.99	1181.24	2616.60
**SNR**	**2.4**	**2.7**	**3.3**

SNR = Average/Standard deviation [McCreery. R.L. Signal-to-Noise in Raman Spectroscopy, in: Raman Spectroscopy for Chemical Analysis, Chemical Analysis, John Wiley & Sons, Ltd, 2005; volume 157, pp. 49–71.]

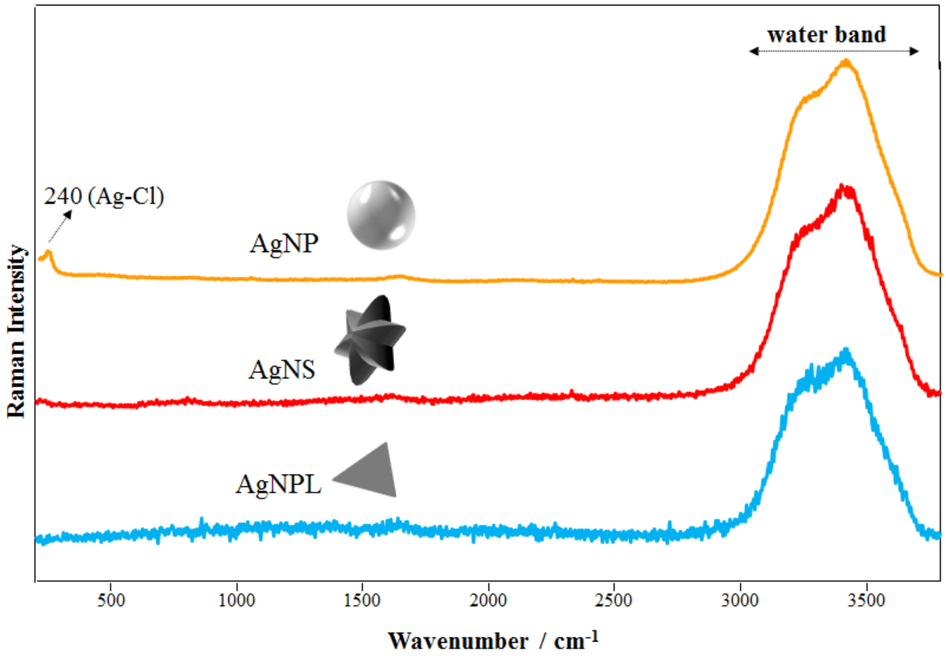

Figure S2. Raman spectra of AgNPs, AgNS and AgNPR colloidal suspensions (as reference).

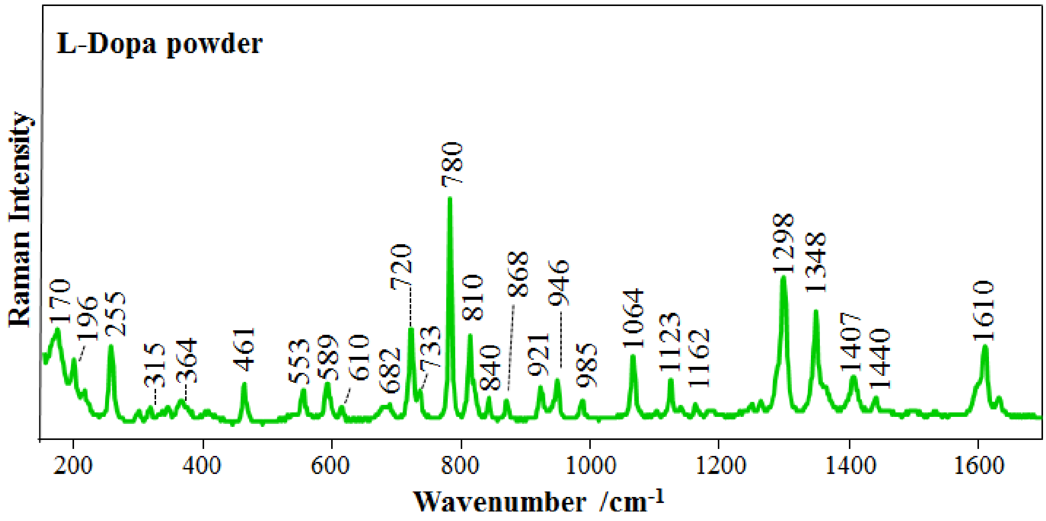

Figure S3. Raman spectrum of L-Dopa powder. Laser line at 633 nm.

Table S2. Assignments of Raman vibrational bands characteristic of L-Dopa powder.

**L-Dopa powder (cm^{-1})**	**Assignments**	**Ref.**
170	C-C-C in-plane bending; C-C-N stretching	[73]
196	C-O-H twisting	[73]
255	C-NH_2 twisting; C-O-H twisting	[73]
315	C-C-N stretching; C-C-O in-plane bending	[73]
364	C-C-N stretching	[73]
461	Ring asymmetric deformation	[73]
553	C-O in-plane bending and C-C stretching of Dopa ring; ring symmetric deformation	[73]
589	C-C in-plane bending of Dopa ring; Dopa ring asymmetric twisting; Dopa ring puckering	[73]
610	C-C-N stretching; C-C-O in-plane bending; C=O rocking	[73]
682	Dopa ring puckering; C-O in-plane bending of Dopa ring	[73]
720	C-C stretching of Dopa ring; C=O in-plane bending; C-C stretching	[73]
733	C-C and C-O stretching	[73]
780	C-H and C-O in-plane bending of Dopa ring	[73]
810	C-O-H twisting; C-N-H in-plane bending	[73]
840	C-H in-plane bending of Dopa ring; C-N stretching; C-C-H in-plane bending	[73]
868	C-H in-plane bending of Dopa ring; Dopa ring puckering	[73]
921	C-C stretching of Dopa ring; C-C stretching	[73]
946	C-C-H in-plane bending; C-N-H in-plane bending	[73, 74]
985	C-N stretching; C-C stretching	[73]
1064	C-O stretching and C-C stretching of Dopa ring; C-O-H in-plane bending	[73]
1123	C-C stretching and C-H in-plane bending of Dopa ring	[73]

1162	C-H in-plane bending; C-C stretching of phenyl ring; O-H out-of-plane	[73,74]
1298	C-C stretching of Dopa ring; C-C-H in-plane bending	[73]
1348	C-C stretching of Dopa ring; O-H out-of-plane bending	[73,75,76]
1407	C-H in-plane bending and C-C stretching of Dopa ring; C-O-H and C-C-H in-plane bending	[73,76]
1440	C-O-H in-plane bending; C-O stretching	[73]
1610	C-C stretching and C-H in-plane bending of Dopa ring	[73]

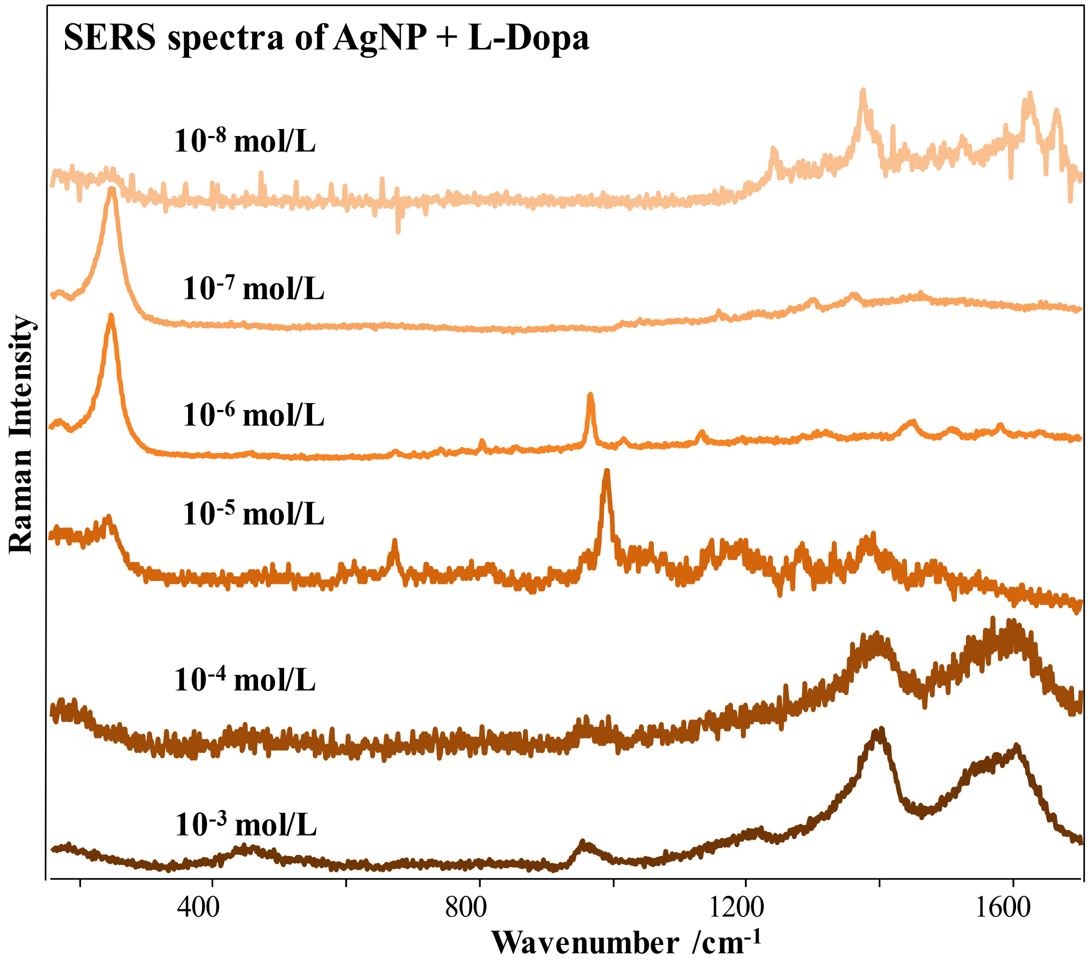

Figure S4. SERS spectra of L-Dopa solutions in colloidal suspension of AgNP at different concentrations (from 10^{-3} to 10^{-8}mol/L). Laser line at 633 nm.

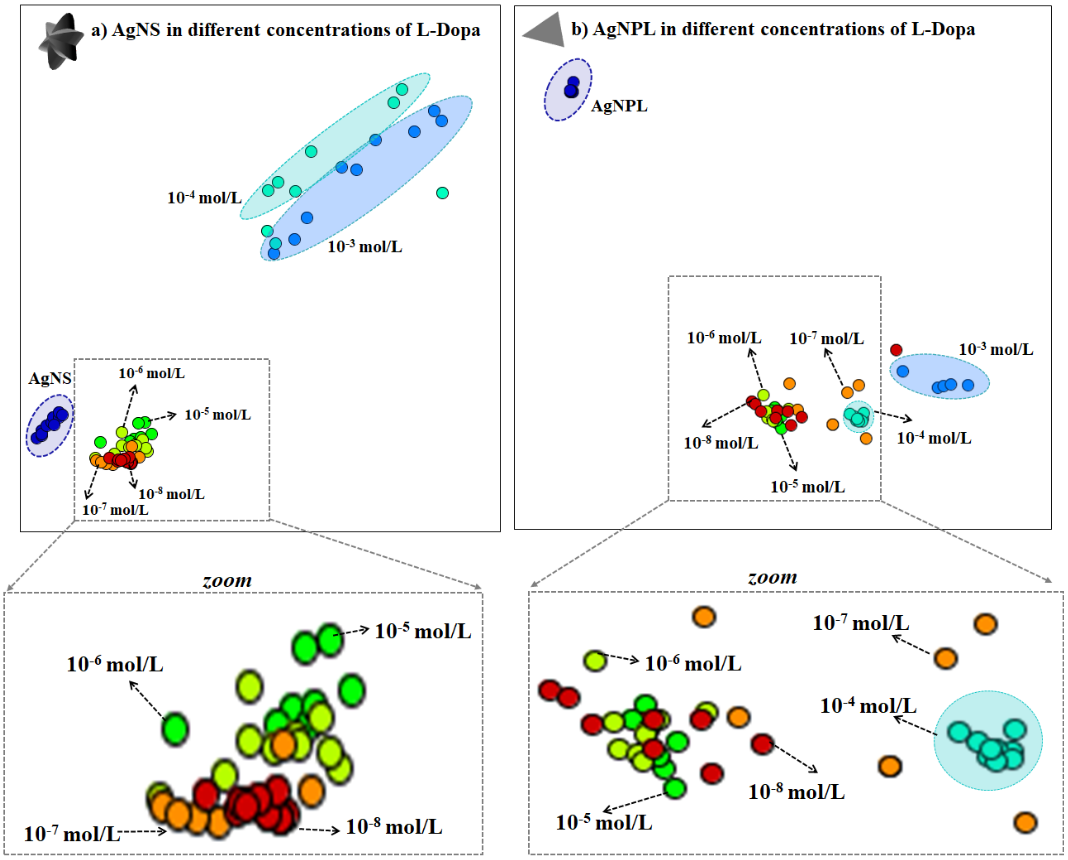

Figure S5. Zoom of the IDMAP multidimensional projection for concentrations of L-Dopa (**a**) down to 10^{-5} mol/L in colloidal suspension of AgNS and (**b**) down to 10^{-4} mol/L in colloidal suspension of AgNPL. Each circle in the plot represents a whole SERS spectrum. The proximity of the circles indicates the similarity between the SERS spectra.

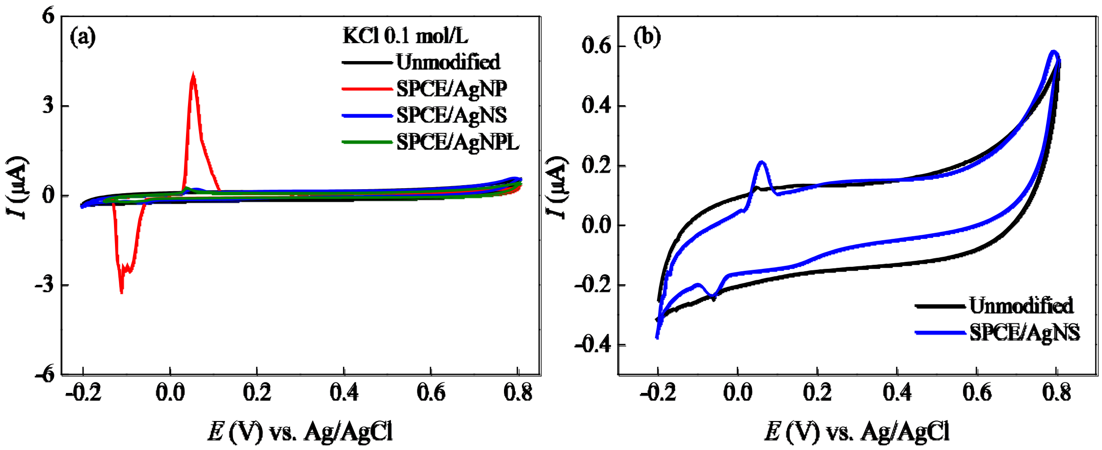

Figure S6. (**a**) Cyclic voltammetry of SPCE unmodified and modified with AgNP, AgNS and AgNPL in 0.1 mol/L KCl solution. (**b**) Cyclic voltammetry of SPCE unmodified and modified with AgNS from Figure S6 (a) for better view. v = 25 mV/s.
